# T100. NICOTINE USE IMPACTS NEGATIVE SYMPTOMS SEVERITY IN SCHIZOPHRENIA

**DOI:** 10.1093/schbul/sby016.376

**Published:** 2018-04-01

**Authors:** Hianna Oliveira, Luccas Coutinho, Cinthia Higuchi, Cristiano Noto, Rodrigo Bressan, Ary Gadelha

**Affiliations:** Universidade Federal de São Paulo

## Abstract

**Background:**

Nicotine use is higher among patients with schizophrenia (50–98%) than in general population (25–30%). This association can reflect a non-specific liability to substance use or specific effects of tobacco on symptoms severity or side effects. Studies about nicotine use and schizophrenia symptoms dimensions are controversial. Some of them showed a relation between severe nicotine use and higher positive symptoms and others presented a correlation between lower negative symptoms and nicotine use. That is why we aimed to verify whether nicotine use is associated with symptoms dimensions in patients with schizophrenia.

**Methods:**

Two hundred and seven outpatients were enrolled from the Programa de Esquizofrenia da Universidade Federal de São Paulo (PROESQ/UNIFESP). Schizophrenia diagnosis was confirmed by Structured Clinical Interview for DSM-IV Axis I Disorders (SCID-I). Dimensional psychopathology was assessed with Positive and Negative Syndrome Scale (PANSS) and Fagerstrom Test for Nicotine Dependence. The PANSS items were grouped in five dimensions: positive, negative, disorganized/cognitive, mood/depression and excitement/hostility. The total score of Fagerstrom Test for Nicotine Dependence was the index used for severity in nicotine dependence. We used Wilcoxon-mann- whitney test to compare the means of PANSS dimensions between nicotine users versus non nicotine use.

**Results:**

The patients mean age was 36.75 (SD 10.648), 69.1% were male, 48.3% reported lifetime tobacco use and 34.3% reported current tobacco use. Lower scores on negative dimension were associated with nicotine use (W = 5642.5, p-value = 0.046, effect size = 0.446). All p-values were corrected by Bonferroni test. Tests that evaluated the relationship between nicotine use and the total PANSS score or other dimensions were not statistically significant.

**Discussion:**

This study shows that nicotine use impacts negative symptoms of schizophrenia. Increase in hepatic metabolism leading to low antipsychotic blood levels has been previously documented in patients with schizophrenia. Thus, the observed results can either indicate effect on primary negative symptoms or indirect effects through reduced D2 blockade caused by lower antipsychotic levels. Future quantitative analyzes and Longitudinal studies may better inform on direction of the association between nicotine dependence and negative symptoms in schizophrenia.

